# The Effect of Adjuvant Radiotherapy on One- and Two-Stage Prosthetic Breast Reconstruction and on Autologous Reconstruction: A Multicenter Italian Study among 18 Senonetwork Breast Centres

**DOI:** 10.1155/2023/6688466

**Published:** 2023-05-09

**Authors:** Andrea Vittorio Emanuele Lisa, Marzia Salgarello, Alessandra Huscher, Fabio Corsi, Daniele Piovani, Federica Rubbino, Stefania Andreoletti, Giovanni Papa, Francesco Klinger, Corrado Tinterri, Alberto Testori, Marta Scorsetti, Paolo Veronesi, Maria Cristina Leonardi, Mario Rietjens, Umberto Cortinovis, Valeria Summo, Emanuele Rampino Cordaro, Pier Camillo Parodi, Paolo Persichetti, Mauro Barone, Giorgio De Santis, Matteo Murolo, Michele Riccio, Angelica Aquinati, Francesco Cavaliere, Nicola Vaia, Giulia Pagura, Erica Dalla Venezia, Franco Bassetto, Vincenzo Vindigni, Luigi Ciuffreda, Maria Alessandra Bocchiotti, Alberto Sciarillo, Nadia Renzi, Graziano Meneghini, Tajna Kraljic, Andrea Loreti, Lucio Fortunato, Valentina Pino, Valeriano Vinci, Marco Klinger

**Affiliations:** ^1^Reconstructive and Aesthetic Plastic Surgery School, Department of Medical Biotechnology and Translational Medicine BIOMETRA-Plastic Surgery Unit, University of Milan, IRCCS Humanitas Research Hospital, Rozzano, Milan, Italy; ^2^Department of Plastic Surgery, Director of the Residency Program of Plastic Surgery, IRCCS Policlinico Gemelli, Università Cattolica del Sacro Cuore, Rome, Italy; ^3^Department of Radiotherapy, Fondazione Poliambulanza “Guido Berlucchi” Hospital, Brescia, Italy; ^4^Breast Unit, Department of Surgery, IRCCS Istituti Clinici Maugeri, Pavia, Italy; ^5^Department of Biomedical and Clinical Sciences, University of Milan, Milan, Italy; ^6^Department of Biomedical Sciences, IRCCS Humanitas University, Pieve Emanuele, Milan, Italy; ^7^IRCCS Humanitas Research Hospital, Rozzano, Milan, Italy; ^8^Laboratory of Molecular Gastroenterology, IRCCS Humanitas Research Hospital, Rozzano, Milan, Italy; ^9^Department of Plastic Surgery, UCO, University of Trieste, Trieste, Italy; ^10^IRCCS Multimedica, Sesto San Giovanni, Milan, Italy; ^11^Breast Unit, IRCCS Humanitas Research Hospital, Rozzano, Milan, Italy; ^12^Radiotherapy and Radiosurgery Department, IRCCS Humanitas Research Hospital, Rozzano, Milan, Italy; ^13^Division of Senology, Department of Oncology and Oncohematology, IEO, IRCCS European Institute of Oncology, University of Milan, Milan, Italy; ^14^Division of Radiotherapy, IEO, IRCCS European Institute of Oncology, Milan, Italy; ^15^Division of Plastic and Reconstructive Surgery, IEO, IRCCS European Institute of Oncology, Milan, Italy; ^16^Department of Plastic Reconstructive Surgery, IRCCS National Cancer Institute, Milan, Italy; ^17^Department of Plastic Surgery, Azienda Sanitaria Universitaria Friuli Centrale (ASUFC), Santa Maria della Misericordia Hospital, Udine, Italy; ^18^Campus BioMedico, University of Rome, Rome, Italy; ^19^Department of Plastic Surgery, Policlinico di Modena, University of Modena and Reggio, Modena, Italy; ^20^Department of Reconstructive Surgery and Hand Surgery, Azienda Ospedaliero Universitaria-Ospedali Riuniti, Ancona, Italy; ^21^Department of Breast Surgery and Integrated Senology Centre, Belcolle Hospital, Viterbo, Italy; ^22^Department of Breast Surgery, AULSS 3 Veneziana, Venice, Italy; ^23^Unit of Plastic Surgery, Department of Neurosciences, University of Padova, Padova, Italy; ^24^Department of Breast Surgery and Breast Unit, Casa Sollievo della Sofferenza Hospital, San Giovanni Rotondo, Foggia, Italy; ^25^Department of Plastic Surgery, Città Della Salute e della Scienza Hospital, Turin, Italy; ^26^Department of Plastic Surgery, ASUGI Cattinara Hospital, Trieste, Italy; ^27^Functional Department Transmural Breast Surgery, AULSS 8 Berica, Vicenza, Italy; ^28^Department of Breast Surgery, AULSS 8 Berica, Vicenza, Italy; ^29^Department of Plastic Surgery, San Giovanni Addolorata Hospital, Rome, Italy; ^30^Department of Breast Surgery and Breast Unit, San Giovanni Addolorata Hospital, Rome, Italy; ^31^Istituto di Clinica Chirurgica, Università Cattolica del Sacro Cuore, Rome, Italy

## Abstract

**Purpose:**

In modern breast cancer treatment, a growing role has been observed for breast reconstruction together with an increase in clinical indications for postmastectomy radiotherapy (PMRT). Choosing the optimum type of reconstructive technique is a clinical challenge. We therefore conducted a national multicenter study to analyze the impact of PMRT on breast reconstruction.

**Methods:**

We conducted a retrospective case-control multicenter study on women undergoing breast reconstruction. Data were collected from 18 Italian Breast Centres and stored in a cumulative database which included the following: autologous reconstruction, direct-to-implant (DTI), and tissue expander/immediate (TE/I). For all patients, we described complications and surgical endpoints to complications such as reconstruction failure, explant, change in type of reconstruction, and reintervention.

**Results:**

From 2001 to April 2020, 3116 patients were evaluated. The risk for any complication was significantly increased in patients receiving PMRT (aOR, 1.73; 95% CI, 1.33–2.24; *p* < 0.001). PMRT was associated with a significant increase in the risk of capsular contracture in the DTI and TE/I groups (aOR, 2.24; 95% CI, 1.57–3.20; *p* < 0.001). Comparing type of procedures, the risk of failure (aOR, 1.82; 95% CI, 1.06–3.12, *p*=0.030), explant (aOR, 3.34; 95% CI, 3.85–7.83, *p* < 0.001), and severe complications (aOR, 2.54; 95% CI, 1.88–3.43, *p* < 0.001) were significantly higher in the group undergoing DTI reconstruction as compared to TE/I reconstruction.

**Conclusion:**

Our study confirms that autologous reconstruction is the procedure least impacted by PMRT, while DTI appears to be the most impacted by PMRT, when compared with TE/I which shows a lower rate of explant and reconstruction failure. The trial is registered with NCT04783818, and the date of registration is 1 March, 2021, retrospectively registered.

## 1. Introduction

In modern breast cancer treatment, the demand for postmastectomy reconstruction is growing. After surgery, for those women who so choose, preserving the breast mound with immediate or delayed reconstruction is crucial to ensure an adequate quality of life [1, 2].

Several reconstruction techniques are available, which are as follows: two-stage tissue expander and implants (TE/I), single-stage direct-to-implant reconstruction (DTI), and autologous tissue grafts.

Choosing the optimum type of reconstructive technique is a clinical challenge depending on patient preference, risks and benefits of each technique, baseline risk factors for reconstruction failure such as high BMI or smoking, and the need for postmastectomy radiation therapy (PMRT) [3].

PMRT is an integral part of breast cancer, reducing local recurrence and improving survival [4].

The evidence to support PMRT is somehow discordant. Guidelines in the United States are represented by the National Comprehensive Cancer Network (NCCN) [5] and the American Society for Radiation Oncology (ASCO) [6, 7].

PMRT is recommended for patients with locally advanced disease (T3 or higher) or with pathologically-involved lymph nodes (N2-N3, N1 under discussion) [8]. The NCCN in particular recommends PMRT for patients with greater than or equal to 4 positive nodes based on level 1 evidence. Furthermore, the NCCN recommends that PMRT should be “strongly considered” in patients with 1 to 3 positive lymph nodes taking into account other risk factors such as age, comorbidities, life-expectancy, the tumor size, the ratio of positive lymph nodes, tumor grade, LVSI, or biologic tumor features (i.e., hormonal receptor status and targetable mutations). These recommendations are in line with recently updated PMRT Guidelines issued by the American Societies of Clinical, Radiation, and Surgical Oncology [5, 6, 9].

PMRT volumes include the chest wall, regional lymph nodes, dissected/nondissected axilla, supraclavicular fossa, and internal mammary nodes [10].

The benefits of PMRT and the growing clinical practice of postmastectomy reconstruction have raised many questions about their optimal integration.

There is a significant gap in the published medical literature regarding the effect of radiotherapy on the three types of reconstruction. In particular, given the extreme variability in clinical approaches, there is no consensus regarding the most appropriate surgical technique or the correct reconstructive timing with respect to radiotherapy [11].

Senonetwork Italia, a nonprofit organization devoted to supporting the quality of multidisciplinary breast cancer (BC) care, has promoted the collection into a multicenter database of data generated by several Italian Breast Centres of the network. The present work aims to analyze the impact of PMRT on breast reconstruction and to compare the three principal types of reconstruction techniques with a view to determining the one associated with the lowest rate of complications in PMRT settings.

## 2. Methods

### 2.1. Sample and Data Collection

We conducted a retrospective case-control multicenter study on women undergoing breast reconstruction in 18 Italian centers from 2001 to 2020 (listed in Supplementary Table 1). The study was submitted for approval to the Ethics Committee of the Humanitas Research Hospital and uploaded to the Clinical Trials database (ClinicalTrials.gov NCT04783818).

Data were collected from 18 Italian Breast Centres and stored in a cumulative database created for this study and managed by the Humanitas Research Hospital.

Inclusion criteria were as follows: female patients, age over 18 years at the time of diagnosis, and patients who had undergone mastectomy and immediate reconstruction with implant in one or two stages or with autologous flaps with or without adjuvant radiotherapy. Exclusion criteria were as follows: patients with prior breast or chest wall irradiation, delayed reconstruction, and patients lost to follow-up.

In the compilation of the database, each sample was considered as a breast and not as a patient; each patient undergoing mastectomy and subsequent bilateral reconstruction was therefore considered as two samples.

The average follow-up period was two years, and patients were monitored weekly in the first month and thereafter every 3–6–12 months.

### 2.2. Reconstructive Procedure and Endpoints

In our study population, we included the following: autologous reconstruction, direct-to-implant reconstruction (DTI), and tissue expander/immediate reconstruction (TE/I).

Patients who underwent reconstruction with latissimus dorsi and implant or expander were included either in the DTI group or the TE/I group.

In order to standardize the study population, in the TE/I group, we decided to exclude patients who received radiotherapy on definitive implant since the majority of patients received radiotherapy during the tissue expander procedure.

In the prosthetic group, we only included retropectoral reconstructions since the prepectoral patients had shorter follow-up. In addition, only a few patients had an acellular dermal matrix reconstruction (ADM) and these were excluded.

Postoperative radiation therapy was prescribed in accordance with national and international guidelines. In all cases, the chest wall was included in irradiation volumes.

For all patients, we described complications and possible surgical endpoints to complications.

We considered the following as mild complications: hematoma, seroma, skin necrosis, abdominal and lumbar pain, liponecrosis, and volume loss. Severe complications included the following: capsular contraction, hernia, flap necrosis, bulging, microvascular complication, and late infection. Since the study is multicenter and each hospital has different scales of assessment, we preferred to evaluate only the presence or the absence of each complication without scales.

We divided infections into “early” and “late,” considering late infections as those occurring 30 or more days from surgery.

We did not grade capsular contracture in order to overcome heterogeneity of evaluation; we considered capsular contracture as “present” or “absent.” The criteria for capsular contraction were as follows: painful sensation, deformed breast or asymmetry of the two breasts, visible thickening of the implant, and visually “nonaesthetic” appearance.

Four different endpoints for reconstructive complications were chosen, which are as follows: reconstruction failure, explant, change in type of reconstruction, and reintervention.

The term “reconstruction failure” refers to patients who did not have any other breast reconstruction after implant or flap removal. The term “explant,” on the other hand, was employed when the prosthesis or flap were removed without contraindication for another reconstructive procedure.

The change in type of reconstruction was reported as referring to the situation where after the explant, the primary reconstruction (DTI, TE/I, or flap) was replaced by or integrated with another type of reconstruction.

Reinterventions included the following: escharotomy in cases of necrosis, seroma, and hematoma drainage, capsular revision or implant substitution, and microvascular revision.

Our primary endpoint is to provide a general overview considering the large numbers of complications and outcomes of the various reconstructive types in relation to PMRT. We believe that for a patient all the aspects greatly impact the reconstruction process and that they should therefore be fully informed about what reconstructive types exist and what are their possible adverse outcomes. The choice should be individualized for each patient and take patient's expectations, a careful evaluation of each technique's pros and cons, and an accurate assessment of patient's baseline risk factors.

### 2.3. Statistical Analysis

We described patients' characteristics at the baseline and treatments received at the baseline, as well as postmastectomy outcomes and complications, in the overall cohort and by type of surgical procedure performed.

We summarized data as medians and interquartile ranges (IQR) or percentages and compared them across groups of patients by using the chi-squared test or the Fischer exact test (categorical data) or the Kruskal–Wallis test (continuous variables) as appropriate.

We investigated the risk of failure, explant and reintervention, as well as the risk of any complication, severe complications, and any infection, according to the type of procedure performed by means of multivariable hierarchical logistic regression.

To investigate the association between PMRT and outcomes or complications, we performed multivariable hierarchical logistic regression analyses and specified a random intercept for each of the 18 recruiting centers. The model was prespecified and included relevant confounders identified by literature screening and expert opinion [11–15]. The analyses were adjusted for age, the BMI, smoking habits, diabetes, other autoimmune diseases, type of chemotherapy, axillary dissection, and type of surgical procedure. Missing data amounted to less than 2% per variable, with the exception of the BMI (13.5%). We considered values as missing at random and performed a complete case analysis. However, for each outcome measure, we performed a regression analysis not including the BMI (Table1 model 1) and a maximally adjusted analysis including the BMI (Table 1 model 2).

We used STATA 17.0 (Stata Corp., College Station, TX, USA) for the statistical analysis. A two-sided*p* value <0.05 was considered statistically significant.

## 3. Results

From 2001 to April 8, 2021, 3116 records were retained for the analysis: 187 autologous, 1227 direct-to-implant, and 1702 tissue expander/immediate reconstructions (Table 2).

Baseline demographic and clinical characteristics of the overall cohort stratified by the surgical procedure are reported in Table 2 and Supplementary Table 2.

The median age of patients was 49 years (IQR, 43–56), with patients undergoing DTI being a median of 3 years younger than those receiving other types of surgical procedures (Table 2). The median BMI was 22.9 (IQR, 20.8–25.3); this value was significantly higher in patients undergoing autologous reconstruction (*p* < 0.001), due to the higher proportion of obese patients in this group (*p* < 0.001, Table 2). Among other risk factors, 14.5% of patients were current smokers, 3.3% had diabetes, and 7.3% had autoimmune disease. Most patients received chemotherapy (61.1%), 46.1% underwent axillary dissection, and 37.5% underwent PMRT. The proportion of patients undergoing these treatments and procedures significantly differed by type of surgical procedure performed (Table 2).

### 3.1. Postsurgical Outcomes and Complications

The key negative postoperative outcomes and complications by type of surgical procedure are reported in Table 3 and Supplementary Table 3. Failure occurred in a small proportion of patients (2.3%), with no significant differences between patients undergoing different types of reconstruction (*p*=0.54).

It should be noted that in irradiated expander, failure accounted for 3.8% of the patients while in irradiated DTI this increased to 5.6%.

Accordingly, the proportion of patients undergoing explant was significantly higher in the DTI group (14.1%; *p* < 0.001, Table 3); taking irradiation into account, we observed an increase in the explant rate comparing the expander and DTI (both irradiated and nonirradiated) which resulted as 12.8% vs. 27% and 4.8 vs. 8%, respectively (Supplementary Table 3).

It should be noted that the proportion of patients needing a reintervention was higher in the TE/I group (19.0%; *p* < 0.001), when compared with both autologous reconstruction and DTI.

The number of patients who needed a change in the type of reconstruction was higher in the group undergoing autologous reconstruction (4.9%; *p* < 0.001, Table 3) and more precisely in the irradiated autologous reconstructions (11.5% against 0% of the nonirradiated autologous group (Supplementary Table 3)).

In 39.1% of the cases, we registered at least one complication, divided as follows: 19.2% severe complications, 10.3% mild complications, 7.0% concomitant mild and severe complications, and 2.6% of unknown or unclassified severity. Infections occurred in 4.6% of cases (*n* = 144) (Table 3).

For implant-based breast reconstruction, the most frequent complication was capsular contracture accounting for 12.5% of patients undergoing DTI and 7.2% in TE/I in nonirradiated patients. Such percentages increased dramatically in irradiated patients up to 51.8% and 46.2%, respectively (Supplementary Table 3).

In the group undergoing autologous reconstruction, the most common severe complications were as follows: presence of flap necrosis of any grade (11.9%), microvascular complications requiring reintervention (6.2%), and bulging (4.0%). While in the groups undergoing DTI and TE/I reconstruction, the most severe complication by far was capsular contracture, occurring, respectively, in 25.0% (*n* = 307) and 22.3% (*n* = 367) of the reconstructions performed (Table 3).

### 3.2. Postmastectomy Radiotherapy as a Risk Factor for Key Outcomes and Complications

The multivariable-adjusted odds ratios (i.e., adjusted for age, the BMI, smoking habits, diabetes, other autoimmune diseases, type of chemotherapy, axillary dissection, and type of surgical procedure performed) between PMRT and key outcomes and complications are reported in Table 1.

Taking into account the above-mentioned confounders, PMRT increased the risk of key negative outcomes including reconstruction failure (aOR, 2.72; 95% CI, 1.39–5.32; *p*=0.004), need for a change in type of reconstruction (aOR, 7.99; 95% CI, 1.36–47.0; *p*=0.021), and explant (aOR, 2.49; 95% CI, 1.49–4.15; *p* < 0.001) but not reintervention (aOR, 1.49; 95% CI, 0.89–2.48; *p*=0.128).

The risk for any complication was significantly increased in patients receiving PMRT (aOR, 1.73; 95% CI, 1.33–2.24; *p* < 0.001), in particular those of severe grade (aOR, 2.17; 95% CI, 1.58–2.97; *p* < 0.001), while mild complications were not significantly affected by irradiation. PMRT was associated with a significant increase in the risk of any infection (aOR, 1.91; 95% CI, 1.06–3.42; *p*=0.030) but not of late infections (aOR, 0.82; 95% CI, 0.28–2.38; *p*=0.713) together with an association with the risk of capsular contracture in the DTI and TE/I groups (aOR, 2.24; 95% CI, 1.57–3.20; *p* < 0.001).

### 3.3. Risk of Negative Outcomes and Complications by Type of Procedure

In adjusted analyses (taking into account PMRT, age, the BMI, smoking habits, diabetes, other autoimmune diseases, type of chemotherapy, and axillary dissection), the risk of failure (aOR, 1.82; 95% CI, 1.06–3.12, *p*=0.030), explant (aOR, 3.34; 95% CI, 3.85–7.83, *p* < 0.001), any complication (aOR, 1.83; 95% CI, 1.43–2.34, *p* < 0.001), and severe complications (aOR, 2.54; 95% CI, 1.88–3.43, *p* < 0.001) were significantly higher in the group undergoing DTI reconstruction as compared to TE/I reconstruction. However, the adjusted odds of reintervention were about 20 times lower in patients with DTI reconstruction (aOR, 0.05; 95% CI, 0.03–0.08, *p* < 0.001) (Table 4).

The group of patients undergoing DTI reconstruction had a significantly higher risk of explant (aOR, 3.80; 95% CI, 1.15–8.03, *p*=0.026) as compared with those who underwent autologous reconstruction, but the adjusted odds of reinterventions were about nine times lower (aOR, 0.11; 95% CI, 0.03–0.43, *p*=0.002). As compared to those with a TE/I reconstruction, those undergoing autologous reconstructions had an increased risk of complications (aOR, 2.27; 95% CI, 1.41–3.67, *p*=0.001) and severe complications (aOR, 2.29; 95% CI, 1.24–4.23, *p*=0.008) (Table 4). There were no significant differences in terms of infections across any of the three groups.

## 4. Discussion

Nowadays, indications for PMRT are increasing together with the rate of breast reconstruction, given an increased awareness of its fundamental role.

In order to guide physicians towards better practice, there is a clear need for guidelines and position statements in the medical-scientific community. In particular, the impact of PMRT on breast reconstruction has been a subject of longstanding research, but a large study population is still needed to confirm the evidence [12, 16, 17].

For this reason, Senonetwork Italia supported this project in order to evaluate the effect of PMRT on breast reconstruction and to compare the three principal types of reconstruction techniques.

The retrospective experience of 18 centers was collected, and the different clinical experiences reported were also collected. In the three different populations, we analyzed the percentage of absolute reconstruction failure, explant, reintervention, and change in type of reconstruction.

Categorizing the reintervention represented a particularly challenging task, since we included in this group general surgical complications such as escharotomy in cases of necrosis, seroma, or hematoma drainage together with implant-related reinterventions and microvascular revisions. Our analysis of the reintervention was aimed at obtaining data regarding the possibility of receiving additional intervention after primary surgery, thus offering patients comprehensive preoperative information.

Our study confirms the negative effect of PMRT on breast reconstruction after adjusting for a consistent set of clinical and surgical confounders. PMRT increased the risk of complication, explant, and reconstruction failure, but interestingly, not of late infection. In addition, our analysis confirmed that radiotherapy is related to a significant increase in the risk for capsular contraction in both DTI and TE/I patients.

In our study population, we collected a great majority of implant-based breast reconstructions (94%) including patients who underwent both latissimus dorsi and implants; such data are consistent with the Italian experience where prosthetic reconstruction is highly prevalent.

Nevertheless, autologous reconstruction has been adopted as a viable option in view of adjuvant radiotherapy, based on the widespread belief in the greater safety of this technique, especially in the case of PMRT [18, 19].

In our population, the absolute failure rate was as low as 2.3%, with no significant differences between type of reconstruction. However, when taking into consideration radiotherapy, autologous reconstruction appeared to be the least impacted if compared with implant-based breast reconstruction.

Our observations, although limited by the reduced percentage of patients, are consistent with more recent studies that confirm the superiority of autologous reconstruction in terms of complication rates and reconstructive failure in cases of PMRT [12, 20–25].

Despite the advantages described above, the autologous approach is characterized by longer intervention and recovery times than prosthetic reconstructions together with morbidity of the donor site [24, 26].

Given the safer profile of autologous reconstruction, the greatest dilemma in prosthetic breast reconstruction is whether to choose DTI or TE/I reconstruction, especially when radiotherapy is administered. According to Sun et al., PMRT can increase implant reconstruction complications and prosthetic reconstruction failure but remains an acceptable option in a multidisciplinary setting [27]. There is a growing debate about which implant-based breast reconstruction is preferable to offer in cases of PMRT [10, 20, 21, 23].

Lin and colleagues argued in favor of DTI, comparing irradiated single-stage breast reconstruction and two-stage breast reconstruction when TE is irradiated after the first stage [28].

Similar considerations are described in the multivariate analysis of Naoum et al. that showed a significant association between TE/I and a higher risk of infection and implant failure, when compared to autologous reconstruction. However, DTI and autologous reconstruction showed comparable outcomes [20, 29].

Unlike Lin and Naoum's reports, in our study, we observed a higher proportion of explant in irradiated DTI when compared with irradiated TE/I.

A more detailed comparison between our analysis and that of Naoum and coworkers reveals that the two analyses differ slightly. In particular, in our investigation, radiotherapy is considered as a confounder, while in Naoum's paper nonirradiated patients are excluded. For this reason, in our analysis, the results are applicable to patients both with and without radiotherapy, as radiotherapy was included in the multivariable model.

We are convinced that our statistical analysis is more consistent with current clinical practice, in which the need for PMRT is not usually known before breast reconstruction, so we aimed to evaluate the best reconstruction procedure considering any clinical variable. Indeed, the statistical adjustment performed on our population, which allowed us to include both PMRT patients and non-PMRT patients, demonstrated a higher power in detecting any difference when compared to the analysis which considered irradiated patients only.

According to our data, TE/I reconstruction appears to be safer when compared to DTI; indeed, in patients who underwent DTI, we observed that complications may lead more frequently to failure and explant, while positioning implants after expander substitution in irradiated fields seems to lead to failure rate being halved.

Mun's metanalysis [30] presented comparable results based on the belief that direct pressure or excess tension on a mastectomy skin flap due to the insertion of a large fixed-volume implant could increase the rate of reconstruction failure and explant, especially in the case of PMRT.

From our results, we are convinced that radiotherapy affects both DTI and TE/I. However, in TE/I breast reconstruction, the impact of radiotherapy is not reflected in an increase in failure or the explant rate but rather in an increased reintervention rate and a consequently greater need for implant substitution or capsular revision.

Although TE/I reconstruction appears to be the safest prosthetic option when PMRT is administered, these observations warrant further evaluation to ascertain the best timing of radiotherapy in relation to expander substitution, percentage of expander filling during radiotherapy, or whether the use of lipofilling during the substitution exerts an impact on final reconstruction failure.

Our analysis further highlights how radiotherapy leads to capsular contracture in both DTI and TE/I reconstruction. Such an observation, based on a well-populated national database, testifies the need for procedures to reduce and treat capsular contracture.

Our study presents certain limitations that warrant consideration. Firstly, it is retrospective in nature. Secondly, although collecting experience from 18 breast centers allowed us to obtain a substantial study population, there is some degree of variability in experience and clinical practice across centers. To overcome this limitation, we employed a multivariable hierarchical logistic regression analysis with a random intercept which took into account the fact that observations belonging to each recruiting center are to a certain degree correlated.

As previously stated, in order to obtain an additional evaluation, we analyzed the proportion of reinterventions including a wide range of different procedures. We are aware that merging different procedures may somewhat mislead in terms of conclusions; nevertheless, considering each single reintervention separately would not have allowed us to draw a meaningful conclusion because of a lack of statistical power.

We are convinced, however, that these data still provide useful information regarding the chance to receive further procedures after the first operation.

Lastly, we were unable to include data regarding ADM usage and prepectoral reconstruction. ADM is widely adopted, especially in studies conducted in the US; however, Italian centres do not routinely use ADM. Italian population sample sizes were therefore too small to provide any meaningful data. Prepectoral reconstruction is being increasingly indicated, thanks to a rapid postoperative recovery, reduction of postoperative pain, and the absence of animation deformity. Italian centers are thus progressively shifting towards prepectoral reconstruction. So far, the follow-up period of studies is still short; nevertheless, we are currently planning to conduct a more extensive, comprehensive, and definitive investigation into the effects of radiotherapy on prepectoral implants.

## 5. Conclusion

Our large multicenter study [31] confirms that autologous reconstruction is the procedure that is least impacted by PMRT. However, it is more invasive, calls for a dedicated microsurgical team, and demonstrates a higher risk of changing the type of reconstruction.

Direct-to-implant reconstruction appears to be procedure that is most impacted by PMRT if compared with the two-stage approach. Our findings show that the TE/I approach appears to be the safer option in PMRT cases.

Nevertheless, further data are needed to elicit the safety profile of two-stage breast reconstruction in PMRT cases, with an analysis of whether filling volume during radiotherapy could affect outcomes or whether autologous fat grafting could improve results.

## Figures and Tables

**Table 1:** Multivariable hierarchical logistic regression analyses investigating the association between postmastectomy radiotherapy and key postsurgical outcomes and complications. Each estimate is adjusted for age, smoking habits, diabetes mellitus, other autoimmune diseases, type of chemotherapy, axillary dissection, and type of surgical procedure performed (autologous reconstruction, direct to implant, or tissue expander/immediate). Estimates in the second column (multivariable model 2) are additionally adjusted for the BMI.

Dependent variable	Radiotherapy			
	Multivariable model 1		Multivariable model 2	
	aOR (95% CI)	*p* value^†^	aOR (95% CI)	*p* value^†^
*Outcomes*				
Failure	2.60 (1.38–4.88)	0.003	2.72 (1.39–5.32)	0.004
Change in type of reconstruction	5.66 (1.20–26.6)	0.028	7.99 (1.36–47.0)	0.021
Explant	2.31 (1.40–3.80)	0.001	2.49 (1.49–4.15)	<0.001
Reintervention	1.24 (0.79–1.94)	0.347	1.49 (0.89–2.48)	0.128
*Complications (all procedures)*				
Any complication	2.00 (1.58–2.54)	<0.001	1.73 (1.33–2.24)	<0.001
Severe complication^1^	2.80 (2.09–3.74)	<0.001	2.17 (1.58–2.97)	<0.001
Mild complication^2^	1.60 (1.18–2.17)	0.003	1.28 (0.91–1.80)	0.162
Hematoma	0.61 (0.33–1.14)	0.120	0.48 (0.23–1.02)	0.056
Seroma	1.82 (1.23–2.70)	0.003	1.49 (0.92–2.41)	0.101
Cutaneous necrosis	0.91 (0.60–1.39)	0.664	0.68 (0.42–1.10)	0.114
Any infection	1.76 (1.09–2.86)	0.022	1.91 (1.06–3.42)	0.030
Late infection	1.18 (0.52–2.64)	0.693	0.82 (0.28–2.38)	0.713
*Complications in DTI and TE/I groups*				
Capsular contraction	3.18 (2.31–4.37)	<0.001	2.24 (1.57–3.20)	<0.001
Implant exposure	2.05 (1.07–3.92)	0.029	1.94 (0.99–3.79)	0.054
Implant rupture	1.31 (0.49–3.54)	0.591	1.85 (0.64–5.36)	0.258

Values are expressed as absolute frequency (percentage) for categorical variables and as median (interquartile range) for continuous variables. Due to the small sample size and number of events in the group undergoing autologous reconstruction, it was not possible to perform the multivariable regressions using as outcomes the following complications occurring exclusively in this group: liponecrosis, volume loss, pain, hernia, flap necrosis, bulging, and microvascular complications. ^†^Chi-squared test for categorical variables; Kruskal–Wallis test for continuous variables. ^1^Severe complications: late infection, capsular contraction, implant exposure, implant rupture, hernia, flap necrosis, bulging, and microvascular complications requiring surgery. ^2^Mild complications: hematoma, seroma, cutaneous necrosis, early infection, abdominal or lumbar pain, liponecrosis, and volume loss. Abbreviations: aOR: adjusted odds ratio; BMI: body mass index; DTI: direct to implant; TE/I: tissue expander/immediate.

**Table 2:** Baseline demographic, clinical characteristics, and treatments of the overall cohort stratified by the surgical procedure.

Variable	Overall cohort (3,119 pts)	Autologous reconstruction (187 pts)	Direct to implant (1,230 pts)	Tissue expander/immediate (1,702 pts)	*p* value^†^
Age (years; *n* = 3,102)	49 (43–56)	50 (45–56)	47 (42–54)	50 (44–58)	<0.001
BMI (kg/m^2^; *n* = 2,698)	22.9 (20.8–25.3)	25.4 (23.1–28.0)	22.0 (20.2–24.0)	23.4 (21.1–26.2)	<0.001
≥30	163 (6.0)	20 (11.8)	28 (2.5)	115 (8.3)	<0.001
Smoking (*n* = 3,077)					
Smokers	447 (14.5)	18 (10.1)	184 (15.3)	245 (14.4)	
Ex-smokers	372 (12.1)	18 (10.1)	144 (12.0)	210 (12.4)	0.32
Nonsmokers	2,258 (73.4)	142 (79.8)	874 (72.7)	1,242 (73.2)	
Diabetes mellitus (*n* = 3,086)	102 (3.3)	6 (3.2)	34 (2.8)	62 (3.6)	0.49
Other autoimmune disorders (*n* = 3,119)	229 (7.3)	14 (7.5)	112 (9.1)	103 (6.1)	0.007
Chemotherapy (*n* = 3,115)					
No	1,213 (38.9)	45 (24.1)	485 (39.6)	683 (40.1)	
Adjuvant	1,273 (40.9)	99 (52.9)	447 (36.4)	727 (42.7)	<0.001
Neoadjuvant	616 (19.8)	43 (23.0)	294 (24.0)	279 (16.4)	
Adjuvant and neoadjuvant	13 (0.4)	0 (0.0)	0 (0.0)	13 (0.8)	
Axillary dissection (*n* = 3,112)	1,435 (46.1)	94 (50.3)	504 (41.1)	837 (49.3)	<0.001
Postmastectomy radiotherapy (*n* = 3,116)	1,167 (37.5)	79 (42.3)	392 (32.0)	696 (40.9)	<0.001

Values are expressed as absolute frequency (percentage) for categorical variables and as median (interquartile range) for continuous variables. ^†^Chi-squared test for categorical variables; Kruskal–Wallis test for continuous variables. Abbreviation: BMI: body mass index.

**Table 3:** Postoperative outcomes and complications by type of surgical procedure performed.

Variable	Overall cohort (3,119 pts)	Autologous reconstruction (187 pts)	Direct to implant (1,230 pts)	Tissue expander/immediate (1,702 pts)	*p* value^†^
*Outcomes*					
Failure (no reconstruction; *n* = 3,113)	72 (2.3)	4 (2.1)	33 (2.7)	35 (2.1)	0.54
Change in type of reconstruction (*n* = 3,041)	30 (1.0)	9 (4.9)	14 (1.2)	7 (0.4)	<0.001
Explant (*n* = 3,119)	265 (8.5)	7 (3.7)	173 (14.1)	85 (5.0)	<0.001
Reintervention (*n* = 3,119)	357 (11.5)	6 (3.2)	28 (2.3)	323 (19.0)	<0.001
*Complications*					
Any complication (*n* = 3,118)	1,218 (39.1)	73 (39.0)	469 (38.1)	676 (39.7)	0.68
Severity of complications (*n* = 3,119)^1^					
No complication	1,900 (60.9)	114 (61.0)	761 (61.9)	1,025 (60.9)	<0.001
Exclusively mild complications	321 (10.3)	33 (17.6)	91 (7.4)	197 (11.6)	
Exclusively severe complications	598 (19.2)	9 (4.8)	253 (20.6)	336 (19.7)	
Mild and severe complications	217 (7.0)	26 (13.9)	94 (7.6)	97 (5.7)	
Severity unknown	83 (2.6)	5 (2.7)	31 (2.5)	47 (2.8)	
Hematoma (*n* = 3,115)	111 (3.6)	14 (7.5)	34 (2.8)	63 (3.7)	0.005
Seroma (*n* = 3,114)	278 (8.9)	6 (3.2)	99 (8.1)	173 (10.2)	0.002
Cutaneous necrosis (*n* = 3,114)	193 (6.2)	28 (15.0)	77 (6.3)	88 (5.2)	<0.001
Any infection (*n* = 3,115)	144 (4.6)	12 (6.4)	43 (3.5)	89 (5.2)	0.041
Timing of infections (*n* = 3,115)					
No infection	2,971 (95.4)	175 (93.6)	1,187 (96.5)	1,609 (94.8)	0.034
Early infection	42 (1.3)	6 (3.2)	8 (0.7)	28 (1.6)	
Late infection	58 (1.9)	3 (1.6)	25 (2.0)	30 (1.8)	
Early and late infection	1 (0.0)	0 (0.0)	0 (0.0)	1 (0.0)	
Timing unknown	43 (1.4)	3 (1.6)	10 (0.8)	30 (1.8)	
Capsular contraction (*n* = 2,875)	674 (23.4)	NA	307 (25.0)	367 (22.3)	0.097
Implant exposure (*n* = 2,928)	69 (2.4)	NA	31 (2.5)	38 (2.2)	0.62
Implant rupture (*n* = 2,928)	30 (1.0)	NA	5 (0.4)	25 (1.5)	0.005
Liponecrosis (*n* = 177)	19 (10.7)	19 (10.7)	NA	NA	NA
Volume loss (*n* = 177)	15 (8.5)	15 (8.5)	NA	NA	NA
Pain (*n* = 177)	4 (2.3)	4 (2.3)	NA	NA	NA
Abdominal pain	4 (2.3)	4 (2.3)	NA	NA	NA
Lumbar pain	0 (0.0)	0 (0.0)	NA	NA	NA
Hernia (*n* = 177)	3 (1.7)	3 (1.7)	NA	NA	NA
Flap necrosis (*n* = 177)			NA	NA	NA
No	156 (88.1)	156 (88.1)	NA	NA	NA
Partial	16 (9.1)	16 (9.1)	NA	NA	NA
Full thickness	5 (2.8)	5 (2.8)	NA	NA	NA
Bulging (*n* = 177)	7 (4.0)	7 (4.0)	NA	NA	NA
Microvascular complications (*n* = 177)^2^	11 (6.2)	11 (6.2)	NA	NA	NA

Values are expressed as absolute frequency (percentage) for categorical variables and as median (interquartile range) for continuous variables. ^†^Chi-squared test for categorical variables; Kruskal–Wallis test for continuous variables. ^1^Mild complications: hematoma, seroma, cutaneous necrosis, early infection, abdominal or lumbar pain, liponecrosis, and volume loss. Severe complications: late infection, capsular contraction, implant exposure, implant rupture, hernia, flap necrosis, bulging, and microvascular complications requiring surgery. ^2^Microvascular complication requiring surgery.

**Table 4:** Multivariable hierarchical logistic regression analyses investigating the association between type of surgical procedure performed and key negative outcomes and complications. Each estimate is adjusted for postmastectomy radiotherapy, age, the BMI, smoking habits, diabetes, other autoimmune diseases, type of chemotherapy, and axillary dissection.

Type of surgical procedure	Failure		Explant		Reintervention		Any complication		Severe complications		Any infection		Change in type of reconstruction	
	aOR (95% CI)	*p*	aOR (95% CI)	*p*	aOR (95% CI)	*p*	aOR (95% CI)	*p*	aOR (95% CI)	*p*	aOR (95% CI)	*p*	aOR (95% CI)	*p*
First model specification^1^														
Autologous reconstruction	Ref.	—	Ref.	—	Ref.	—	Ref.	—	Ref.	—	Ref.	—	Ref.	—
DTI reconstruction	1.75 (0.58–5.27)	0.323	3.80 (1.49–9.63)	0.005	0.11 (0.03–0.43)	0.002	0.81 (0.51–1.28)	0.360	1.11 (0.61–2.02)	0.740	0.60 (0.25–1.41)	0.242	0.12 (0.03–0.49)	0.004
TE/I reconstruction	0.96 (0.32–2.86)	0.940	1.14 (0.44–2.91)	0.792	2.20 (0.59–8.17)	0.240	0.44 (0.27–0.71)	0.001	0.44 (0.24–0.80)	0.008	0.77 (0.32–1.83)	0.552	0.05 (0.10–0.27)	<0.001
Second model specification^1^														
DTI reconstruction	Ref.	—	Ref.	—	Ref.	—	Ref.	—	Ref.	—	Ref.	—	Ref.	—
Autologous reconstruction	0.57 (0.19–1.73)	0.323	0.26 (0.10–0.67)	0.005	9.09 (2.30–35.9)	0.002	1.24 (0.78–1.98)	0.360	0.90 (0.50–1.64)	0.740	1.67 (0.71–3.93)	0.242	8.59 (2.02–36.5)	0.004
TE/I reconstruction	0.55 (0.32–0.94)	0.030	0.30 (0.22–0.41)	<0.001	20.0 (12.1–33.0)	<0.001	0.55 (0.43–0.70)	<0.001	0.39 (0.29–0.53)	<0.001	1.28 (0.74–2.23)	0.378	0.45 (0.16–1.26)	0.129
Third model specification^1^														
TE/I reconstruction	Ref.	—	Ref.	—	Ref.	—	Ref.	—	Ref.	—	Ref.	—	Ref.	—
Autologous reconstruction	1.04 (0.35–3.11)	0.940	0.88 (0.34–2.25)	0.792	0.45 (0.12–1.69)	0.240	2.27 (1.41–3.67)	0.001	2.29 (1.24–4.23)	0.008	1.30 (0.55–3.08)	0.552	19.1 (3.75–97.6)	<0.001
DTI reconstruction	1.82 (1.06–3.12)	0.030	3.34 (2.46–4.54)	<0.001	0.05 (0.03–0.08)	<0.001	1.83 (1.43–2.34)	<0.001	2.54 (1.88–3.43)	<0.001	0.78 (0.45–1.36)	0.378	2.23 (0.79–6.27)	0.129

^1^We have specified different baseline levels for the variable indicating the type of surgical procedure performed and have alternatively selected a different group as the one corresponding to the baseline risk. This allowed to show all the formal comparisons across groups. Abbreviations: aOR: adjusted odds ratio; BMI: body mass index; DTI: direct to implant; TE/I: tissue expander/immediate.

## Data Availability

The database used to support the findings of this study were supplied by the nonprofit organization Senonetwork group; requests for access to these data should be made to Daniele Piovani.

## Abbreviations

ADM: Acellular dermal matrix
DTI: Direct-to-implant
PMRT: Postmastectomy radiotherapy
TE/I: Tissue expander/implants.

## Supplementary Materials

Supplementary Materials Supplementary Table 1: Italian centers that participated in the creation of the Senonetwork database. Supplementary Table 2: baseline demographic, clinical characteristics, and treatments of the overall cohort stratified by the surgical procedure and postmastectomy radiotherapy. Supplementary Table 3: postoperative outcomes and complications stratified by the surgical procedure and postmastectomy radiotherapy.
