# Supplementary material for: The Effect of Adjuvant Radiotherapy on One- and Two-Stage Prosthetic Breast Reconstruction and on Autologous Reconstruction: A Multicenter Italian Study among 18 Senonetwork Breast Centres
Source: Breast J. 2023 May 9;2023:6688466. doi: 10.1155/2023/6688466 (PMC10188256; doi:10.1155/2023/6688466)
Supplement: Supplementary Materials — Supplementary Table 1: Italian centers that participated in the creation of the Senonetwork database. Supplementary Table 2: baseline demographic, clinical characteristics, and treatments of the overall cohort stratified by the surgical procedure and postmastectomy radiotherapy. Supplementary Table 3: postoperative outcomes and complications stratified by the surgical procedure and postmastectomy radiotherapy. [file 6688466.f1.zip › Supplementary Table 1.docx]

**Supplementary Table 1** Italian centers that participated in the creation of the Senonetwork database

| **Senonetwork** |
| --- |
| - ICH - Humanitas Research Hospital - IEO - Istituto Europeo di Oncologia - INT- Istituto Nazionale dei Tumori - Policlinico Universitario Fondazione Agostino Gemelli di Roma - Presidio Ospedaliero Universitario “S. Maria della Misericordia” di Udine - Policlinico Universitario Campus Bio-Medico - Azienda Ospedaliera-Universitaria di Modena - Ospedali riuniti di Ancona - Ospedale di Belcolle di Viterbo - Fondazione Poliambulanza Brescia - IRCCS Maugeri di Pavia - Ospedale dell’Angelo di Mestre - Azienda Ospedaliera di Padova - IRCCS Casa Sollievo della Sofferenza di San Giovanni Rotondo - A.O.U. Città della Salute e della Scienza di Torino - Azienda ospedaliera Universitaria Ospedali Riuniti di Trieste - AULLS 8 Berica - Azienda Ospedaliera San Giovanni Addolorata – Roma |
