# Supplementary material for: The Effect of Adjuvant Radiotherapy on One- and Two-Stage Prosthetic Breast Reconstruction and on Autologous Reconstruction: A Multicenter Italian Study among 18 Senonetwork Breast Centres
Source: Breast J. 2023 May 9;2023:6688466. doi: 10.1155/2023/6688466 (PMC10188256; doi:10.1155/2023/6688466)
Supplement: Supplementary Materials — Supplementary Table 1: Italian centers that participated in the creation of the Senonetwork database. Supplementary Table 2: baseline demographic, clinical characteristics, and treatments of the overall cohort stratified by the surgical procedure and postmastectomy radiotherapy. Supplementary Table 3: postoperative outcomes and complications stratified by the surgical procedure and postmastectomy radiotherapy. [file 6688466.f1.zip › Supplementary Table 2.docx]

**Supplementary Table 2.** Baseline demographic, clinical characteristics and treatments of the overall cohort and stratified by surgical procedure and post-mastectomy radiotherapy.

| **Variable** | ***Autologous reconstruction***  (187 pts) | | ***Direct to implant***  (1,227 pts) | | ***Tissue expander/immediate***  (1,702 pts) | | **Overall cohort**† |
| --- | --- | --- | --- | --- | --- | --- | --- |
|  | PMRT | NO PMRT | PMRT | NO PMRT | PMRT | NO PMRT |  |
| Number of patients (n=3,116) | 79 (42.3) | 108 (57.7) | 392 (31.9) | 835 (67.9) | 696 (40.9) | 1,006 (59.1) | 3,116 |
| Age (years; n=3,102) | 50 (45‒59) | 50.5 (45‒56) | 49 (43‒56) | 47 (41‒53) | 49 (43‒57) | 50 (44‒58) | 49 (43‒56) |
| BMI (kg/m^2^; n=2,698) | 26.5 (24.0‒28.4) | 25.0 (23.0‒27.1) | 22.4 (20.6‒24.2) | 22.0 (20.1‒23.7) | 23.7 (21.0‒26.6) | 23.2 (21.1‒25.8) | 22.9 (20.8‒25.3) |
| ≥30 | 11 (15.3) | 9 (9.2) | 17 (4.6) | 11 (1.4) | 54 (9.2) | 61 (7.6) | 163 (6.0) |
| Follow-up (years; n=2,832) | 2.6 (0.9‒3.4) | 2.2 (0.5‒2.8) | 2.8 (2.0‒4.0) | 2.4 (1.9‒3.3) | 2.3 (1.5‒3.4) | 2.4 (1.6‒3.2) | 2.4 (1.6‒3.3) |
| Smoking (n=3,077) |  |  |  |  |  |  |  |
| Smokers | 12 (16.4) | 6 (5.7) | 57 (15.2) | 127 (15.4) | 114 (16.5) | 131 (13.0) | 447 (14.5) |
| Ex-smokers | 5 (6.9) | 13 (12.4) | 53 (14.1) | 91 (11.1) | 105 (15.2) | 105 (10.5) | 372 (12.1) |
| Non-smokers | 56 (76.7) | 86 (81.9) | 265 (70.7) | 606 (73.5) | 473 (68.3) | 769 (76.5) | 2,258 (73.4) |
| Diabetes mellitus (n=3,086) | 5 (6.3) | 1 (0.9) | 9 (2.3) | 25 (3.1) | 31 (4.5) | 31 (3.1) | 102 (3.3) |
| Other autoimmune disorders (n=3,119) | 8 (10.1) | 6 (5.6) | 31 (7.9) | 81 (9.7) | 43 (6.2) | 60 (6.0) | 229 (7.3) |
| Chemotherapy (n=3,115) |  |  |  |  |  |  |  |
| No | 8 (10.1) | 37 (34.3) | 41 (10.5) | 444 (53.4) | 139 (20.0) | 554 (54.1) | 1,213 (38.9) |
| Adjuvant | 46 (58.2) | 53 (49.1) | 225 (57.4) | 219 (26.4) | 372 (53.5) | 355 (35.3) | 1,273 (40.9) |
| Neoadjuvant | 25 (31.7) | 18 (16.6) | 126 (32.1) | 168 (20.2) | 175 (25.1) | 104 (10.3) | 616 (19.8) |
| Adjuvant and neoadjuvant | 0 (0.0) | 0 (0.0) | 0 (0.0) | 0 (0.0) | 10 (1.4) | 3 (0.3) | 13 (0.4) |
| Axillary dissection (n=3,112) | 61 (77.2) | 33 (30.6) | 322 (82.1) | 182 (21.9) | 545 (78.3) | 292 (29.1) | 1,435 (46.1) |

Values are expressed as absolute frequency (percentage) for categorical variables and as median (interquartile range) for continuous variables.

# Abbreviation

BMI, body mass index; PMRT, post-mastectomy radiotherapy.
