# Supplementary material for: The Effect of Adjuvant Radiotherapy on One- and Two-Stage Prosthetic Breast Reconstruction and on Autologous Reconstruction: A Multicenter Italian Study among 18 Senonetwork Breast Centres
Source: Breast J. 2023 May 9;2023:6688466. doi: 10.1155/2023/6688466 (PMC10188256; doi:10.1155/2023/6688466)
Supplement: Supplementary Materials — Supplementary Table 1: Italian centers that participated in the creation of the Senonetwork database. Supplementary Table 2: baseline demographic, clinical characteristics, and treatments of the overall cohort stratified by the surgical procedure and postmastectomy radiotherapy. Supplementary Table 3: postoperative outcomes and complications stratified by the surgical procedure and postmastectomy radiotherapy. [file 6688466.f1.zip › Supplementary Table 3.docx]

**Supplementary Table 3.** Post-operative outcomes and complications stratified by surgical procedure and post-mastectomy radiotherapy.

| **Variable** | ***Autologous reconstruction***  (187 pts) | | ***Direct to implant***  (1,227 pts) | | ***Tissue expander/immediate***  (1,702 pts) | | **Overall cohort**  (3,116 pts) |
| --- | --- | --- | --- | --- | --- | --- | --- |
|  | ***PMRT*** | ***NO PMRT*** | ***PMRT*** | ***NO PMRT*** | ***PMRT*** | ***NO PMRT*** |  |
| **Outcomes** |  |  |  |  |  |  |  |
| Failure (no reconstruction; n=3,113) | 1 (1.3) | 3 (2.8) | 22 (5.6) | 11 (1.3) | 26 (3.8) | 9 (0.9) | 72 (2.3) |
| Change in type of reconstruction (n=3,038) | 9 (11.5) | 0 (0.0) | 10 (2.7) | 4 (0.5) | 7 (1.1) | 0 (0.0) | 30 (1.0) |
| Explant (n=3,113) | 4 (5.1) | 3 (2.8) | 106 (27.0) | 67 (8.0) | 88 (12.8) | 48 (4.8) | 316 (10.2) |
| Reintervention (n=3,119) | 3 (3.8) | 3 (2.8) | 17 (4.3) | 11 (1.3) | 253 (36.4) | 70 (7.0) | 357 (11.5) |
| **Complications** |  |  |  |  |  |  |  |
| Any complication (n=3,118) | 37 (46.8) | 36 (33.3) | 253 (64.5) | 215 (25.8) | 409 (58.9) | 267 (26.5) | 1,218 (39.1) |
| Severity of complications (n=3,119)^1^ |  |  |  |  |  |  |  |
| No complication | 42 (53.1) | 72 (66.7) | 139 (35.5) | 620 (74.3) | 286 (41.1) | 739 (73.5) | 1,900 (60.9) |
| Exclusively mild complications | 16 (20.3) | 17 (15.7) | 22 (5.6) | 69 (8.3) | 62 (8.9) | 135 (13.4) | 321 (10.3) |
| Exclusively severe complications | 3 (3.8) | 6 (5.6) | 170 (43.4) | 83 (9.9) | 256 (36.8) | 80 (8.0) | 598 (19.2) |
| Mild and severe complications | 16 (20.3) | 10 (9.3) | 52 (13.3) | 42 (5.0) | 73 (10.5) | 24 (2.4) | 217 (7.0) |
| Severity unknown | 2 (2.5) | 3 (2.8) | 9 (2.3) | 21 (2.5) | 19 (2.7) | 28 (2.8) | 83 (2.6) |
| Hematoma (n=3,115) | 7 (8.9) | 7 (6.5) | 13 (3.3) | 21 (2.5) | 24 (3.5) | 39 (3.9) | 111 (3.6) |
| Seroma (n=3,114) | 2 (2.5) | 4 (3.7) | 47 (12.0) | 52 (6.2) | 86 (12.4) | 87 (8.7) | 278 (8.9) |
| Cutaneous necrosis (n=3,114) | 18 (22.8) | 10 (9.3) | 26 (6.6) | 51 (6.1) | 35 (5.0) | 53 (5.3) | 193 (6.2) |
| Any infection (n=3,115) | 9 (11.4) | 3 (2.8) | 18 (4.6) | 25 (3.0) | 48 (6.9) | 41 (4.1) | 144 (4.6) |
| Timing of infections (n=3,115) |  |  |  |  |  |  |  |
| No infection | 70 (88.6) | 105 (97.2) | 374 (95.4) | 810 (97.0) | 644 (92.5) | 965 (95.9) | 2,971 (95.4) |
| Early infection | 4 (5.1) | 2 (1.9) | 3 (0.8) | 5 (0.6) | 17 (2.4) | 11 (1.1) | 42 (1.3) |
| Late infection | 2 (2.5) | 1 (0.9) | 12 (3.1) | 13 (1.6) | 15 (2.2) | 15 (1.5) | 58 (1.9) |
| Early and late infection | 0 (0.0) | 0 (0.0) | 0 (0.0) | 0 (0.0) | 1 (0.1) | 0 (0.0) | 1 (0.0) |
| Timing unknown | 3 (3.8) | 0 (0.0) | 3 (0.8) | 7 (0.8) | 19 (2.7) | 15 (1.5) | 47 (1.5) |
| Capsular contraction (n=2,875) | NA | NA | 203 (51.8) | 104 (12.5) | 295 (46.2) | 72 (7.2) | 674 (23.4) |
| Implant exposure (n=2,928) | NA | NA | 15 (3.8) | 16 (1.9) | 26 (3.8) | 12 (1.2) | 69 (2.4) |
| Implant rupture (n=2,928) | NA | NA | 2 (0.5) | 3 (0.4) | 12 (1.7) | 13 (1.3) | 30 (1.0) |
| Liponecrosis (n=177) | 9 (12.3) | 10 (9.6) | NA | NA | NA | NA | 19 (10.7) |
| Volume loss (n=177) | 11 (15.1) | 4 (3.9) | NA | NA | NA | NA | 15 (8.5) |
| Pain (n=177) | 2 (2.7) | 2 (1.9) | NA | NA | NA | NA | 4 (2.3) |
| Abdominal pain | 2 (2.7) | 2 (1.9) | NA | NA | NA | NA | 4 (2.3) |
| Lumbar pain | 0 (0.0) | 0 (0.0) | NA | NA | NA | NA | 0 (0.0) |
| Hernia (n=177) | 1 (1.4) | 2 (1.9) | NA | NA | NA | NA | 3 (1.7) |
| Flap necrosis (n=177) |  |  | NA | NA | NA | NA |  |
| No | 60 (82.2) | 96 (92.3) | NA | NA | NA | NA | 156 (88.1) |
| Partial | 10 (13.7) | 6 (5.8) | NA | NA | NA | NA | 16 (9.1) |
| Full-thickness | 3 (4.1) | 2 (1.9) | NA | NA | NA | NA | 5 (2.8) |
| Bulging (n=177) | 1 (1.4) | 6 (5.8) | NA | NA | NA | NA | 7 (4.0) |
| Microvascular complications (n=177)^2^ | 6 (8.2) | 4 (4.8) | NA | NA | NA | NA | 11 (6.2) |

Values are expressed as absolute frequency (percentage) for categorical variables and as median (interquartile range) for continuous variables.

# Footnotes

^1^ Mild complications: hematoma, seroma, cutaneous necrosis, early infection, abdominal or lumbar pain, liponecrosis, volume loss. Severe complications: late infection, capsular contraction, implant exposure, implant rupture, hernia, flap necrosis, bulging, microvascular complications requiring surgery.

^2^ Microvascular complication requiring surgery.
